# PML‐mediated nuclear loosening permits immunomodulation of mesenchymal stem/stromal cells under inflammatory conditions

**DOI:** 10.1111/cpr.13566

**Published:** 2023-10-20

**Authors:** Yunpeng Chu, Zishan Jiang, Zheng Gong, Xiaocao Ji, Mengting Zhu, Qianwen Shang, Pixia Gong, Lijuan Cao, Yongjing Chen, Peishan Li, Changshun Shao, Yufang Shi

**Affiliations:** ^1^ The Third Affiliated Hospital of Soochow University, State Key Laboratory of Radiation Medicine and Protection, Institutes for Translational Medicine Soochow University Medical College Suzhou China

## Abstract

Nuclear configuration plays a critical role in the compartmentalization of euchromatin and heterochromatin and the epigenetic regulation of gene expression. Under stimulation by inflammatory cytokines IFN‐γ and TNF‐α, human mesenchymal stromal cells (hMSCs) acquire a potent immunomodulatory function enabled by drastic induction of various effector genes, with some upregulated several magnitudes. However, whether the transcriptional upregulation of the immunomodulatory genes in hMSCs exposed to inflammatory cytokines is associated with genome‐wide nuclear reconfiguration has not been explored. Here, we demonstrate that hMSCs undergo remarkable nuclear reconfiguration characterized by an enlargement of the nucleus, downregulation of LMNB1 and LMNA/C, decondensation of heterochromatin, and derepression of repetitive DNA. Interestingly, promyelocytic leukaemia‐nuclear bodies (PML‐NBs) were found to mediate the nuclear reconfiguration of hMSCs triggered by the inflammatory cytokines. Significantly, when PML was depleted, the immunomodulatory function of hMSCs conferred by cytokines was compromised, as reflected by the attenuated expression of effector molecules in hMSCs and their failure to block infiltration of immune cells to lipopolysaccharide (LPS)‐induced acute lung injury. Our results indicate that the immunomodulatory function of hMSCs conferred by inflammatory cytokines requires PML‐mediated chromatin loosening.

## Graphical abstract

Our study reveals that MSCs derived from human adipose tissue exhibit cellular nuclear decondensation, reduced heterochromatin, thinning of the nuclear lamina, and increased expression of inflammatory regulatory factors in response to inflammatory factors stimuli. Significant inhibition of the changes in MSC nuclear architecture caused by inflammatory factors can be achieved by siRNA‐mediated interference with the formation of PML‐NBs.

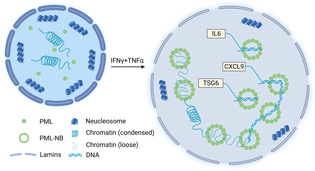

## 1 INTRODUCTION

Chromatin accessibility determines cell lineage identity, fate, and response to environmental and developmental cues.^1^, ^2^, ^3^, ^4^ Eukaryotic DNA wrapped around histones is highly compressed in the nucleus. According to the degree of chromatin condensation, chromatin can be divided into euchromatin and heterochromatin. Constitutive heterochromatin is a fundamental nuclear architecture essential for genome stability, mitosis, transposons silencing, and gene expression regulation.^2^, ^3^, ^4^, ^5^ The status of chromatin condensation is closely related to gene expression. In general, heterochromatin is considered to be transcriptionally inactive.

Mesenchymal stromal/stem cells (MSCs) reside in almost every tissue and play critical roles in tissue homeostasis and regeneration.^6^, ^7^, ^8^ They are indispensable components during tumour development and metastasis.^9^, ^10^, ^11^ MSCs expanded in vitro are well documented to have therapeutic effects in a wide range of autoimmune diseases and hyper‐inflammatory disorders via their immunomodulatory function.^12^, ^13^, ^14^ Significantly, their immunomodulatory properties can be significantly stimulated by inflammatory cytokines, which are accompanied by drastic changes in the transcriptome of hMSCs.^15^, ^16^ Some genes encoding chemokines were even up‐regulated several magnitudes. It is reasonable to assume that such drastic changes in transcriptome in response to inflammatory cytokines got to be associated with nuclear modifications, however, this has not been addressed in the literature.

Promyelocytic leukaemia—nuclear bodies (PML‐NBs) are an archetype of membrane‐less organelles concentrating proteins at discrete sites within the nucleoplasm.^17^, ^18^ In response to stress, they form spheres of ∼0.1–1 μm in diameter in most mammalian nuclei. PML was well‐documented to drive cellular senescence.^19^, ^20^, ^21^, ^22^, ^23^ In recent years, studies have shown that PML‐NBs may participate in the regulation of chromatin dynamics through phase separation.^24^, ^25^ PML‐NBs can accumulate at specific gene loci and coordinate DNA methylation and chromatin remodelling.^18^, ^26^, ^27^ In lymphocytes derived from patients with ICF (Immune Deficiency, Centromere and Facial Abnormality) Syndrome, PML was shown to form giant nuclear bodies with HP1 and hypomethylated satellite DNAs at pericentric heterochromatin regions.^28^ In the PML‐deficient HP1 PML body, the reconstructed protein cannot accumulate around the satellite DNA.

Previously, our team has demonstrated that hMSCs can exhibit powerful immune regulation ability under the combined stimulation of inflammatory factors such as IFNγ, TNFα, and IL‐1, among which IFNγ is indispensable. PML is a typical downstream gene of IFNγ, and IFNγ can significantly up‐regulate the expression of PML.^29^, ^30^ We found that hMSCs stimulated by inflammatory factors could effectively attenuate LPS‐induced acute lung injury or imiquimod‐induced psoriasis‐like inflammation through producing tumour necrosis factor (TNF) stimulating gene‐6 (TSG‐6), a potent immunosuppressive molecule.^31^, ^32^ In addition to TSG‐6, a drastic changes in the transcriptome and secretome of hMSCs have been observed following stimulation of inflammatory factors.

We speculate that these massive expression changes in hMSCs may have been enabled by nuclear reconfiguration. We therefore examined the level of heterochromatic protein HP1a in hMSCs after inflammatory factors stimulation and found it to be decreased significantly in response to inflammatory stimuli. Meanwhile, we found that nuclei were enlarged considerably, the expression of major nuclear lamina proteins LMNB1 and LMNA/C decreased, and the heterochromatin became decondensed after the stimulation by inflammatory factors. In addition, the number of PML‐NBs significantly increased after stimulation of inflammatory factors. PML knockdown significantly attenuated nuclear expansion in hMSCs after inflammatory factors stimulation and inhibited decondensation of heterochromatin. The expression levels of effector molecules by which hMSCs exert immunoregulatory function were also reduced after PML was depleted. Importantly, in an LPS‐induced acute lung injury (ALI) model, the thereapeutic efficacy of hMSCs lacking PML was greatly compromised. Promoting nuclear loosening in response to inflammatory cytokines, PML facilitates transcriptomic changes governing hMSCs' immunomodulatory capacity. Interestingly, the massive nuclear reconfiguration is reversible upon removal of inflammatory cytokines.

## 2 MATERIALS AND METHODS

### 2.1 Cell isolation and culture

hMSCs were obtained from the adipose tissue of lipoaspirate samples following the protocols approved by the Ethics Committee of Soochow University.^33^ The cells were maintained in DMEM/F12 supplemented with 10% FBS, 100 U/mL penicillin/streptomycin solution, and 10 ng/mL bFGF cultured in 10 cm dishes at 37°C in a humidified atmosphere with 5% CO2. The medium was changed every 3 days. Cells at passage 3–9 starting from the primary cells were used for the described experiments. Mouse bone marrow‐derived MSCs, U4A, Hep G2 and EA. Hy926 cell lines cultured in DMEM‐LOW supplemented with 10% FBS, 100 U/mL penicillin/streptomycin solution.

### 2.2 In vitro transfection of siRNA

hMSCs (1.2 × 10^5^ cells/well in 6 well plates) or (2.5 × 10^4^ cells/well in 24 well plates) were seeded in the medium were co‐cultured with siRNA for 24 h, then the medium was removed and then stimulated with inflammatory factors, and the cells were collected for Western blotting, Q‐PCR and immunofluorescence.

### 2.3 Quantitative real‐time PCR

Total RNA was extracted using RNAfast 2000 (Fastagen) and reversely transcribed into cDNA using a ReverTra Ace quantitative polymerase chain reaction (qPCR) RT Kit (TOYOBO Life Science) according to the manufacturer's protocol. qPCR was carried out using SYBR Green Master Mix (4472920, Thermo Fisher Scientific). Primers used are listed in Table S1. The relative mRNA levels of genes were calculated by the 2^−ΔΔCt^ method, using β‐actin as the internal control. Each averaged experimental gene expression sample was compared to the mean in the control sample, which was set to 1.

### 2.4 Western blotting analysis

Equal amounts of protein lysate samples were resolved on sodium dodecyl sulfate‐polyacrylamide gel electrophoresis and then transferred onto polyvinylidene fluoride membranes. After blocking with 5% bovine serum albumin in tris‐buffered saline containing 0.1% Tween‐20 for 2 h, the membranes were probed overnight at 4°C with respective primary antibodies. Membranes were washed three times and incubated with horseradish peroxidase‐conjugated secondary antibody for 1 h at room temperature. Signals were visualized by chemiluminescent substrate (Pierce Biotechnology, Rockford, IL) with a Super sensitive automatic imaging analysis system (Protein simple FluorChem HD2).

### 2.5 Proliferation assay by flow cytometry

hMSCs were stimulated with 10 ng/mL TNFa and IFNγ for 24 h, and EdU (Beyotime) was added into the medium and incubated for 2 h before the cells were fixed and stained. To test the role of PML in hMSCs proliferation, hMSCs were transfected with PML‐specific small interfering RNAs. The transfections were performed using the interferin transfection reagent (Polyplus Transfection) according to the manufacturer's protocol. Nonsilencing siRNA was used to control for any effect of siRNA and the transfection reagent. After 24 h, hMSCs were washed with PBS, cultured in a new complete medium, and stimulated with IFN‐γ and TNF‐α for another 24 h. EdU was added into the medium and incubated for 2 h before the cells were fixed and stained.

### 2.6 Cell cycle analysis by flow cytometry

Cells were trypsinized, washed in PBS, and fixed in 80% methanol at −20°C for at least 2 h. Methanol was removed by centrifugation at 2000*g* for 2 min, and cells were stained with propidium iodide (50 μg/mL) in the presence of RNase A (50 μg/mL) in PBS for 30 min at 37°C. DNA content analysis was performed using the Cytoflex Flow Cytometer (Beckman Coulter). Data were analysed using the Flow Jo software (Version 9.6.2, Tree Star Inc.).

### 2.7 Immunofluorescence

Isolated lung tissues were fixed in 4% paraformaldehyde, embedded in paraffin, cut into 4‐μm‐thick sections, and stained with haematoxylin and eosin. Infiltrating neutrophils were revealed by immunofluorescence using anti‐mouse Ly‐6G/Ly‐6C (Gr‐1) antibody (BioLegend) followed by Alexa 488‐conjugated‐goat anti‐rabbit IgG (Abcam) secondary antibody.

hMSCs were fixed with 4% paraformaldehyde for 15 min, then washed with PBS and permeated with 0.5% Triton X‐100 (V900502‐100ML, Sigma) in PBS for 15 min. The nuclei were stained with Hoechst 33324 (H3570, Thermo Fisher Scientific). The antibodies against Vimentin (ab92547, abcam), HP1α (ab109028, abcam), Lamin B1 (ab16048, abcam), Lamin A/C (ab8984, abcam), H3K9Me2/3(5327, CST), SP100 (ab167605, abcam), and PML (ab179466 and ab96051, abcam) were used as primary antibodies. Secondary antibodies were Alexa 488‐conjugated‐goat anti‐rabbit IgG (Abcam) and Alexa 555‐conjugated‐goat antimouse IgG (Thermo Fisher Scientific). Images were taken by a laser‐scanning confocal microscope (Leica TCS SP8, Leica, Germany).

### 2.8 Cell viability test

Cells were incubated in 10% CCK‐8 (Beyotime, China) diluted in a standard culture medium at 37°C until the visual colour conversion occurred. Each sample's absorbance (450 nm) was analysed by a microplate reader (Cytation5, Bio Tek).

### 2.9 Murine model of LPS‐induced ALI

Six to eight‐week BALB/c female mice were purchased from Beijing Vital River Laboratory Animal Technology Co. Ltd. (Beijing, China) and treated with either 20 μg LPS (Sigma‐Aldrich) from Escherichia coli (serotype 0111:B4) in 50 μL saline or an equal volume of saline as vehicle control, using intranasal insufflation while anaesthetised with tribromoethanol (Sigma‐Aldrich). Human hMSCs (2.5 × 10^5^ in 200 μL PBS) were administered intravenously after LPS administration, while PBS was injected as a control. Mice were euthanized 48 h after LPS exposure. To obtain BAL fluid, lungs were washed three times with 1 mL PBS through i.t. cannulae. The total cell number in BAL fluid was counted, and neutrophil numbers were determined by immunofluorescence staining and flow cytometry. Cells were pre‐incubated with anti‐CD16/CD32 (eBioscience) to block Fc receptors and then stained with anti‐CD45, anti‐CD11b, anti‐Ly‐6G (clone 1A8) for 20 min. Neutrophils were identified by the phenotype CD45 + CD11b + Ly‐6G+. All procedures were approved and conducted under the Guideline for the Institutional Animal Care and Use Committee of Soochow University.

### 2.10 Statistical analysis

Statistical analysis was performed using GraphPad Prism 6 software. Results of multiple observations are presented as means ± SD. Differences between two groups were assessed using an unpaired Student's *t*‐test; for multivariate data analysis, group differences were evaluated using a one‐way analysis of variance with Tukey comparisons; a value *of p* < 0.05 was considered significant.

## 3 RESULTS

### 3.1 Inflammatory cytokines inhibit the proliferation of hMSCs

**FIGURE 1:**
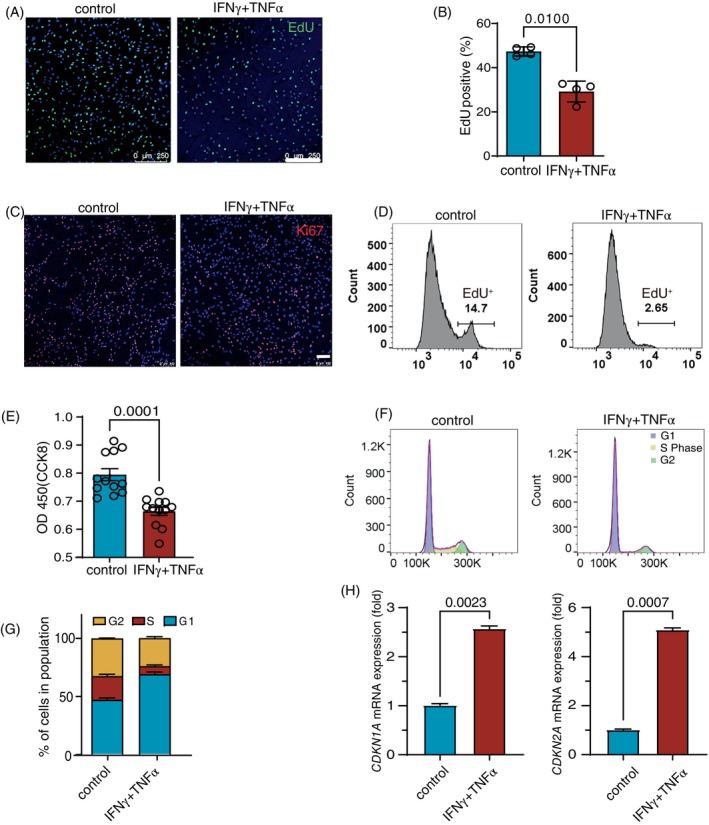
Inflammatory cytokines inhibit the proliferation of hMSCs. (A) Immunostaining of EdU in IFNγ and TNFα pretreated or control hMSCs. Scale bar, 250 μm. (B)The percentage of EdU was analysed with ImageJ, and the calculated data are shown as the mean ± SDs, *n* = 4. Two‐tailed *t*‐test. (C) Immunostaining of Ki67 in IFNγ and TNFα pretreated or control hMSCs. Scale bar, 100 μm. (D) Flow cytometry analysis of EdU in IFNγ and TNFα pretreated or control hMSCs. (E) CCK8 analysis of cell viability in IFNγ and TNFα pretreated or control hMSCs. *n* = 12. Data are presented as the mean ± SDs, a two‐tailed *t*‐test. (F) Effect of IFNγ and TNFα on the cell cycle in hMSC cells. Flow cytometry analysis of EDU in IFNγ and TNFα pretreated or control hMSCs. Representative flow cytometry image. (G) Distribution of cell cycle for hMSCs after treatment with IFNγ and TNFα for 24 h, two‐way ANOVA. (H) Quantitative RT‐PCR analysis of Cyclin‐Dependent Kinase Inhibitor in hMSCs. Two‐tailed t‐test.

Upon stimulation by inflammatory factors, hMSCs undergo drastic changes in global gene expression to exert a potent immunomodulatory effect. We speculate that this functional shift to immunomodulation may compromise other properties such as proliferation. Inflammatory factors are known to arrest the cell cycle and promote cellular senescence of cancer cells.^34^ We assessed hMSC proliferation after exposure to INFγ and TNFα. We first examined the proliferation of hMSCs by EdU incorporation and Ki67 immunofluorescence and the cell viability by CCK8. Immunofluorescence results showed that the proportions of EdU‐positive and Ki67‐positive cells in hMSCs significantly decreased under the stimulation of the inflammatory factors (Figure 1A–C). Flow cytometry further confirmed the reduction in the proportion of EdU‐positive cells in hMSCs treated with the inflammatory cytokines (Figure 1D). CCK8 assay also showed that the cell viability of hMSCs was reduced considerably after stimulation with inflammatory cytokines (Figure 1E). We next examined cell cycle distribution of hMSCs after stimulation with the inflammatory cytokines. The results showed that the fraction of cells in the S phase of hMSCs decreased, whereas the fraction of cells in the G1/G0 phase increased after stimulation by inflammatory cytokines (Figure 1F,G). We also examined the expression of cell cycle regulatory genes *CDKN1A* and *CDKN2A* and found them to be significantly up‐regulated upon inflammatory stimulation (Figure 1H). Together, these results indicate that the proliferation of MSCs is reduced upon exposure to the two cytokines.

### 3.2 Inflammatory cytokines induce nuclear enlargement and lamin loss in hMSCs

**FIGURE 2:**
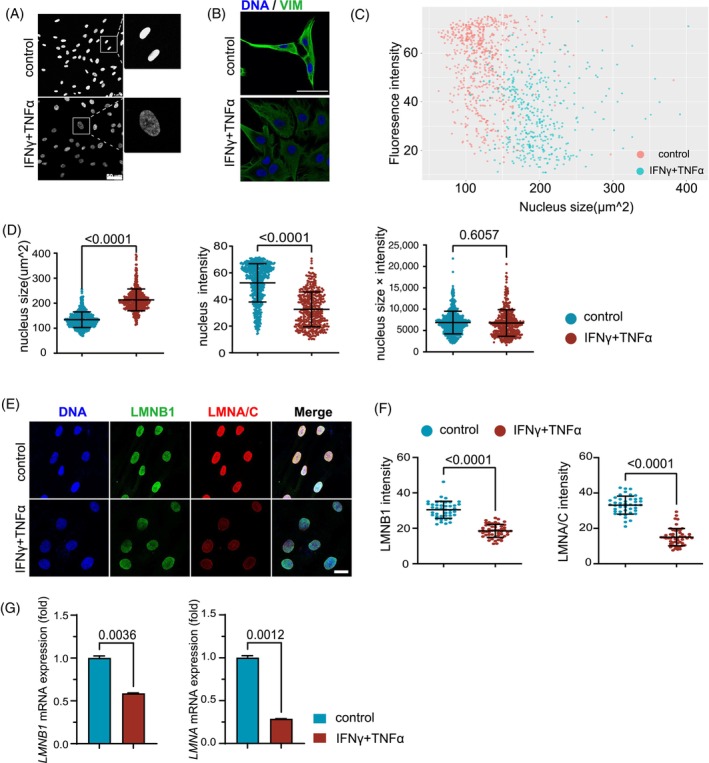
The inflammatory cytokines enlarge nuclei in hMSCs. (A)Chromatin structure of hMSCs shown by Hoechst 33342 staining of the nucleus. Scale bar, 50 μm. (B) Immunostaining of vimentin of hMSCs after treatment with IFNγ and TNFα, Scale bar, 50 μm. (C) Image J was used to score the nuclear size and fluorescence intensity of each cell in the Image, and dot plots were made with the nuclear size as abscissa and fluorescence intensity as ordinate; the red dots show the control group, and the cyan dots show IFNγ and TNFα pretreated group. (D) Values of nuclear Hoechst staining intensity and Hoechst size, and their production, two‐tailed *t*‐test. (E) Immunofluorescence analyses of LMNB1 and LMNA/C expression in control hMSCs and IFNγ and TNFα pretreated hMSCs at P5. Scale bar, 20 μm. (F) Image J was used to score fluorescence intensity of LMNB1 and LMNA/C two‐tailed *t*‐test. (G) hMSCs were treated as in (B), and then the *LMNB1*, *LMNA* mRNA transcripts were examined, two‐tailed *t*‐test.

The results shown above suggest that hMSCs are not merely impaired in their propagation, but might have entered a senescence‐like state. The same cytokines were shown to drive cancer cells into cellular senescence.^34^ Senescent cells are generally accompanied by cellular enlargement and altered heterochromatin.^35^ We speculated that hMSCs stimulated by the inflammatory cytokines might need to loosen their chromatin to facilitate the massive increase in global transcription. We therefore examined whether the cytokines induced morphological changes in hMSCs. The nuclei were drastically enlarged and the chromatin was significantly lossened (Figure 2A). Like senescent cells, hMSCs turned to a more spread polygonal shape with multiple antennae after stimulation with inflammatory factors, as revealed by immunofluorescence staining of vimentin (Figure 2B). By setting the nuclear size as the abscissa and the fluorescence intensity of the nucleus as the ordinate, the hMSCs stimulated by the inflammatory factors and the control cells apparently fell into two distinct groups (Figure 2C). Quantitative analysis showed that nuclei were enlarged in size but the fluorescence intensity decreased significantly after inflammatory stimulation (Figure 2D). The nuclear lamina, which consists of A‐and B‐type lamins, are the main components of the nuclear envelope and determine the shape and size of the nucleus. Loss of LMNB1 has been documented to occur in various types of senescent cells and aging tissues and promote aging^36^, ^37^, ^38^, ^39^ We examined the expression of LMNB1 and LMNA/C by immunofluorescence, and found that both LMNB1 and LMNA/C were downregulated significantly after inflammatory stimulation (Figure 2E,F). Q‐PCR assay produced consistent results (Figure 2G). Together, these results indicated that in response to TNFα and IFNγ, hMSCs were significantly transformed in their nuclear morphology and exhibited senescence‐like phenotypes.

### 3.3 Inflammatory cytokines decondense the heterochromatin in hMSCs

**FIGURE 3:**
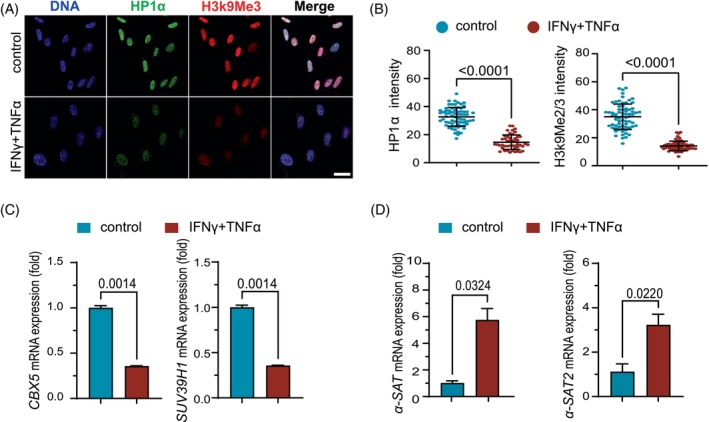
The inflammatory cytokines decondense heterochromatin in hMSCs. (A) Immunofluorescence analyses of HP1α and H3K9Me2/3 expression in control hMSCs and IFNγ and TNFα pretreated hMSCs at P5. Scale bar, 20 μm. (B) Distribution of fluorescence intensity of HP1α and H3K9Me2/3 obtained with Image J, a two‐tailed *t*‐test. (C) hMSCs were treated as in (A), and *CBX5* and *SUV39H1* mRNA levels were examined, two‐tailed *t*‐test. (D) Quantitative RT‐PCR analysis of centromeric repetitive element transcripts in hMSCs after being treated with IFNγ and TNFα for 24 h, two‐tailed *t*‐test.

A decrease in heterochromatinization occurs in senescent cells.^40^, ^41^ We also noted a reduction in the protein level of the primary heterochromatin markers HP1a and H3K9Me2/3 in hMSCs exposed to inflammatory cytokines (Figure 3A,B). *CBX5* and *SUV39H1*, which encodes HP1ɑ and catalyses H3K9Me2/3 respectively, were also downregulated at the transcriptional level (Figure 3C). Previous studies have shown that the loss of H3K9me3 led to an increased transcription of α‐Satellite (α‐Sat) and Satellite 2 (Sat2) from H3K9me3‐enriched centromeric loci in senescent hMSCs.^35^ Quantitative PCR (qPCR) indeed showed that the transcripts of α‐Sat and Sat2 in hMSCs were significantly up‐regulated upon inflammatory stimulation (Figure 3D). Together, these findings indicated that heterochromatin became decondensed in hMSCs in response to the inflammatory cytokines.

### 3.4 PML mediates inflammatory cytokines‐induced nuclear enlargement in hMSCs

**FIGURE 4:**
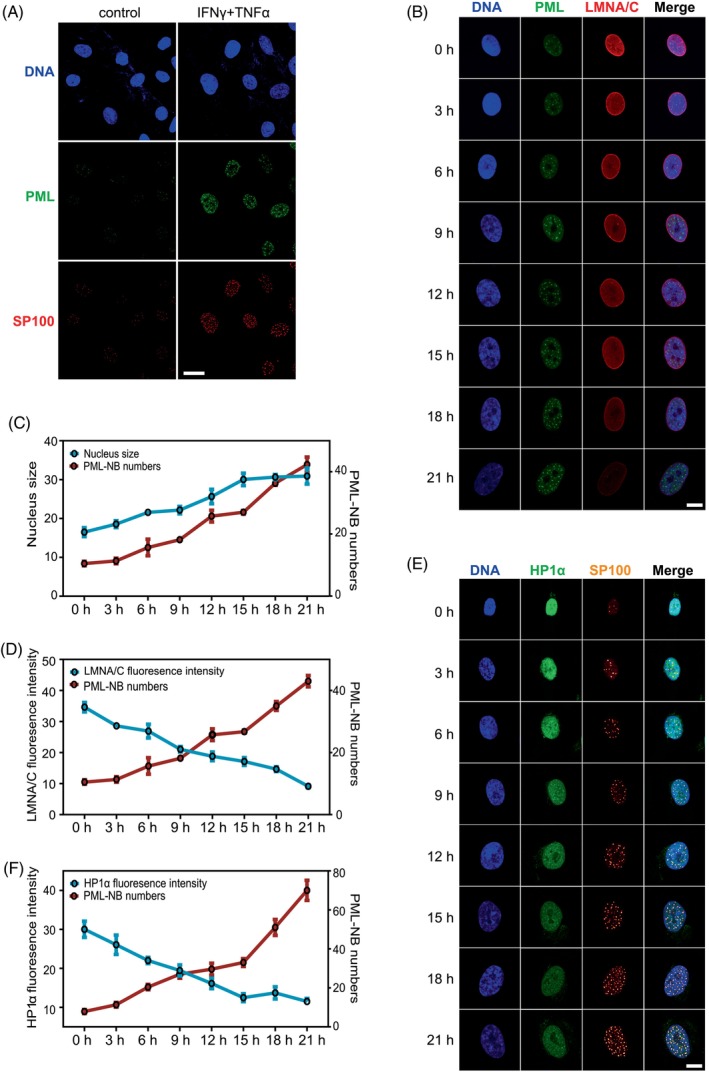
Formation of PML‐NBs associates with changes in nuclear configuration in hMSCs. (A) Immunofluorescence analyses of PML and SP100 expression in control hMSCs and IFNγ and TNFα pretreated hMSCs at P5. Scale bar, 20 μm. (B) Immunofluorescence detection of PML and LMNA/C at different time points in control hMSCs and IFNγ and TNFα pretreated hMSCs at P5. Scale bar, 10 μm. (C,D,F) Changes in nuclear size, LMNA/C and HP1a fluorescence intensities relative to PML‐NB numbers. (E) Immunofluorescence detection of HP1a and SP100 at different time points in control hMSCs and IFNγ and TNFα pretreated hMSCs at P5. Scale bar, 10 μm.

**FIGURE 5:**
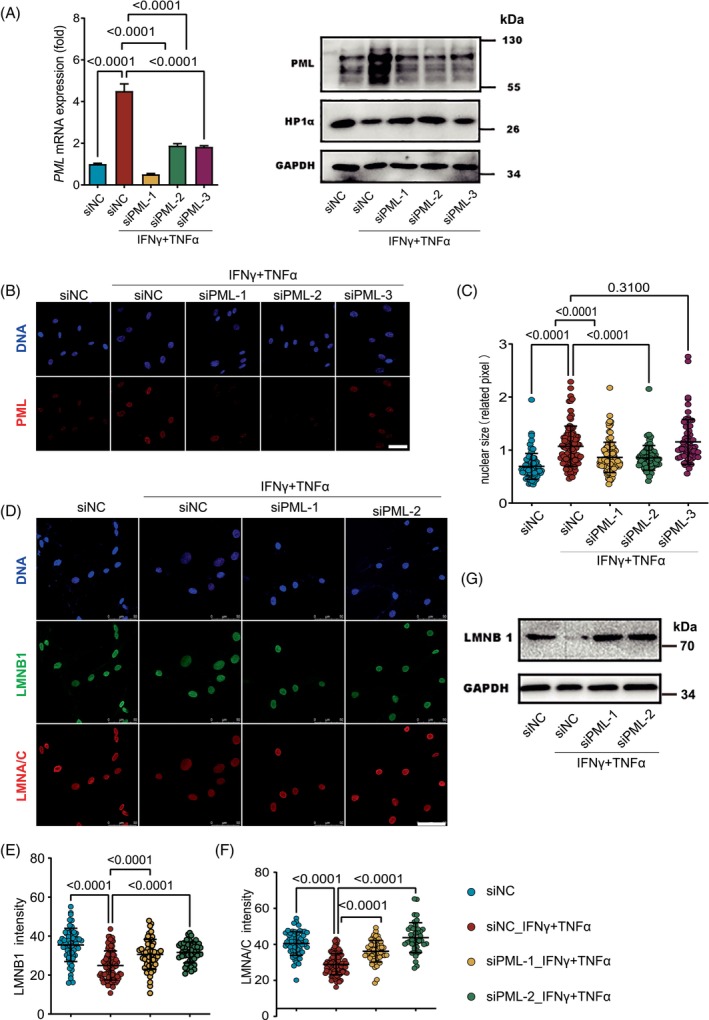
PML‐NBs are required for nuclear reconfiguration in cytokine‐stimulated hMSCs. (A) Left, quantitative RT‐PCR analysis of siRNA knockdown efficiency of *PML* transcripts in hMSCs and right, Western blotting results. (B) Immunofluorescence analyses of PML‐NB after PML knockdown. Scale bar, 50 μm. (C) Distribution of nuclear sizes. (D) Immunofluorescence detection of LMNB1 and LMNA/C after PML knockdown. Scale bar, 50 μm. (E,F) Distribution of LMNB1 and LMNA/C fluorescence intensities after PML knockdown. (G) Western blotting analysis of LMNB1 expression.

PML participates in various biological processes, including the regulation of heterochromatin, by forming nuclear bodies through phase separation.^42^, ^43^ We used immunofluorescence to examine the expression of PML and SP100, a chaperone protein in PML‐NB that can label PML‐NB, in hMSCs after inflammatory stimulation. The results showed that the number and fluorescence intensity of PML‐NBs in hMSCs were strikingly induced by inflammatory stimulation (Figure 4A). We next examined the correlation between the formation of PML‐NB and nuclear size, and the levels of LMNA/C and HP1a in hMSCs at different time points after inflammatory stimulation. We observed that the number of PML‐NBs increased with the duration of treatment, and the sizes of the nuclei also exhibited a positive correlation with the number of PML‐NBs (Figure 4B,C). The intensity of LMNA/C was negatively correlated with the number of PML‐NBs (Figure 4D). The intensity of heterochromatin marker protein HP1a gradually decreased with the increasing number of PML‐NBs (Figure 4E,F). To explore whether the formation of PML‐NBs mediated the nuclear reconfiguration of hMSCs in an inflammatory environment. We designed three siRNA sequences to knock down the expression of PML. The three siRNAs could significantly inhibit the expression of PML, at both mRNA and protein levels (Figure 5A). Interestingly, the immunofluorescence results showed that siPML‐1 or siPML‐2, but not siPML‐3, effectively reduced the number of PML‐NBs upon the inflammatory cytokine stimulation (Figure 5B). We measured the sizes of nuclei of hMSCs and found that siPML‐1 and siPML‐2 each effectively inhibited the nuclear enlargement induced by inflammatory factors, while siPML‐3 was ineffective (Figure 5C). We next determined the expression levels of nuclear laminin LMNA/C and LMNB1 by immunofluorescence staining upon inflammatory factor stimulation. The results showed that whereas LMNA/C and LMNB1 were downregulated significantly after cytokine stimulation, inhibiting the formation of PML‐NBs by siPML abolished the nuclear enlargement and attenuated the decline of LMNA/C and LMNB1 in hMSCs (Figure 5E–G). These results show that formation of PML‐NBs is required for the cytokine‐induced nuclear enlargement in hMSCs.

### 3.5 PML mediates inflammatory cytokines‐induced decondendation of heterochromatin in hMSCs

**FIGURE 6:**
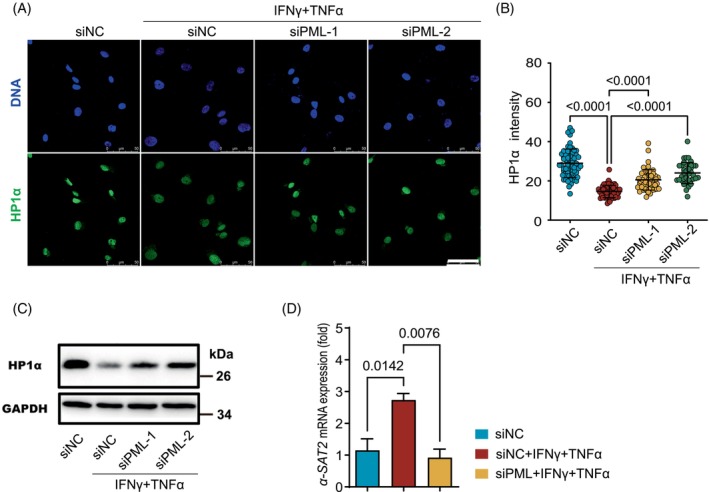
PML mediates heterochromatin decondensation in cytokine‐stimulated in hMSCs. (A) Immunofluorescence analysis of HP1a expression after PML knockdown and then treated with IFNγ and TNFα at P5. Scale bar, 50 μm. (B) Distribution of fluorescence intensities of HP1a, one‐way ANOVA. (C) Western blotting analysis of HP1α expression (D) Quantitative RT‐PCR analysis of *α‐SAT2* expression after PML knockdown and then treated with IFNγ and TNFα, one‐way ANOVA.

Given that PML‐NBs regulates the dynamics of heterochromatin in MEFs,^18^, ^43^ We next explored if blocking the formation of PML‐NBs would alter the HP1a amount and distribution in hMSCs. While stimulation with the cytokines led to decreased heterochromatinization and reduced HP1a expression in hMSCs, this effect was abolished when PML was depleted, as revealed by immunoflourence staining (Figure 6A–C). We examined the expression of a‐SAT2, the repetitive centromeric DNA associated with heterochromatin, and found it to be significantly up‐regulated after inflammatory stimulation, but knockdown of PML could effectively inhibit the up‐regulation of a‐SAT2(Figure 6D). Together, these findings demonstrate that PML‐NBs mediate the decondensation of heterochromatin in hMSCs under an inflammatory environment.

We next determined whether the nuclear expansion caused by the cytokines is unique to human MSCs. We treated four additional cell types or cell lines, including mouse MSCs, with the two cytokines and examined the changes in nuclear configuration. As shown in the Figure S1, all of them exhibited nuclear loosening comparable to what was observed in human MSCs. Increased PML‐NB formation was also observed in these cells.

### 3.6 Formation of PML‐NBs is required for the anti‐inflammatory efficacy of hMSCs in acute lung injury

**FIGURE 7:**
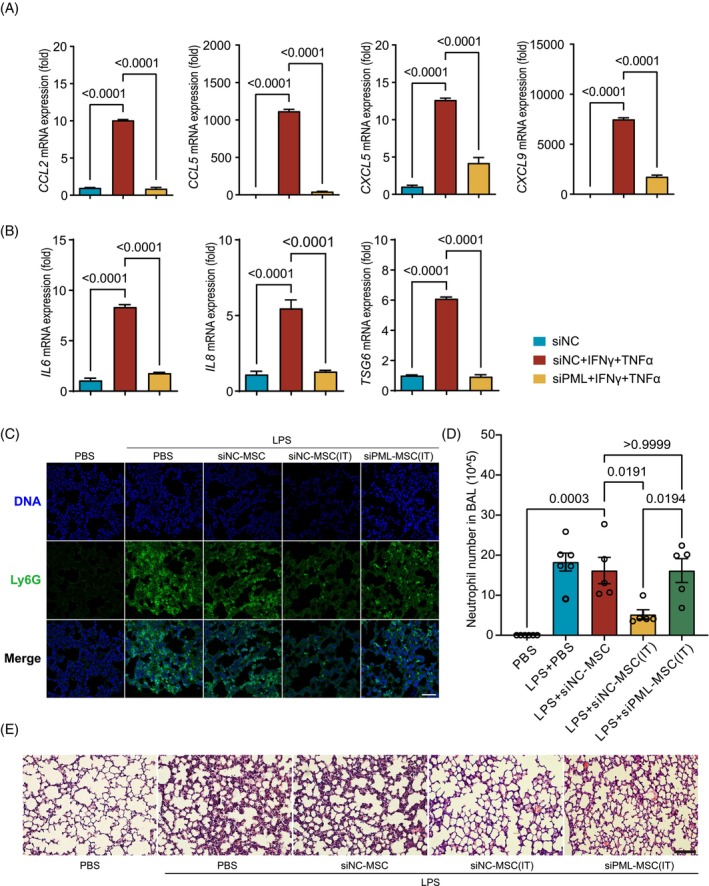
PML insufficiency impairs the therapeutic effect of hMSCs on a mouse model of acute lung injury. (A, B) Quantitative RT‐PCR analysis of chemokine, cytokinesis and TSG6 expression after PML knockdown was treated with IFNγ and TNFα, one‐way ANOVA. (C) hMSCs were transfected with PML siRNA (PML‐KD) or control (ctrl) scramble siRNA. Cells were treated with IFN‐γ and TNF‐α (I + T, 10 ng/mL each)for 24 h. Acute lung injury was induced by LPS administration. PML‐KD hMSCs or control cells (2.5 × 10^5^) were pretreated with IFN‐γ and TNF‐α for 48 h and injected i.v. into Balb/c mice 4 and 28 h after LPS administration. After 2 days, BAL fluid was obtained, and the total cell number was determined. Immunofluorescence analyses of Ly6G positive cells in Lung. Scale bar, 50 μm. (D) Neutrophil cell number in BAL fluid was determined by flow cytometry. Bars indicate mean ± SEM. one‐way ANOVA. (E) H/E staing, Scale bar, 100 μm.

The loose or compact state of chromatin is crucial for the regulation of gene expression. Since the formation of PML‐NBs led to nuclear exapnsion and heterochromatin decondensation in hMSCs stimulated by inflammatory factors, we determined whether the dramatic upregulation of cytokines, chemokines and other effectors of immunomodulation in hMSCs upon stimulation with inflammatory factors requires PML. The results showed that knockdown of PML remarkably blocked the up‐regulation of those immunomodulatory molecules induced by IFN‐γ and TNF‐α (Figure 7A,B). We next examined whether PML‐NBs are required for hMSCs to alleviate acute lung injury (ALI). Accordingly, hMSCs were pretreated with IFN‐γ and TNF‐α in the presence of siRNA to knock down PML, and their therapeutic effects were evaluated. As expected, LPS significantly induced neutrophil infiltration into the lung in the ALI model. While hMSCs that were not pretreated with IFN‐γ and TNF‐α had no effect on the infiltration of neutrophils in the ALI model, hMSCs pretreated with inflammatory factors significantly inhibited the infiltration of neutrophils. However, the augmented efficacy of hMSCs on ALI conferred by IFN‐γ and TNF‐α was abolished when PML was depleted (Figure 7C,D). Together, the above results indicate that PML‐NBs are crucial for hMSCs to acquire the immune regulatory function conferred by inflammatory cytokines.

### 3.7 Cytokines‐induced nuclear loosening is reversible

**FIGURE 8:**
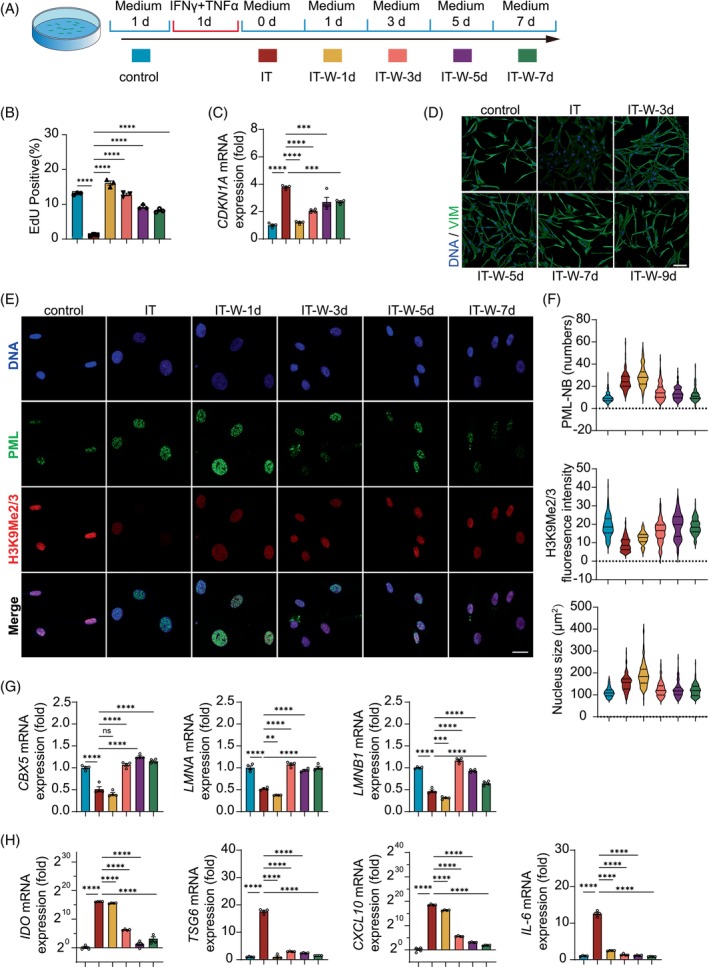
PML‐mediated nuclear changes are reversible. (A) Experimental scheme: hMSCs grown in culture medium served as the control group, while hMSCs stimulated with IFNγ and TNFα (10 ng/mL) for 1 day were designated as the IT group. After 1 day of stimulation, the hMSCs were washed off IT and maintained in in culture medium for 1, 3, 5, and 7 days, respectively, and were referred to as the IT‐W‐1d, IT‐W‐3d, IT‐W‐5d, and IT‐W‐7d groups. After the corresponding treatments, the cells were collected for experiments. (B) Flow cytometry analysis of EdU in IFNγ and TNFα stimulation and withdrawal in hMSCs. (C) Quantitative RT‐PCR analysis of *CDKN1A* expression. (D) Immunostaining of vimentin of hMSCs after IFNγ and TNFα stimulation and withdrawal, Scale bar, 100 μm. (E) Immunofluorescence analyses of PML and H3K9Me2/3 expression in hMSC, Scale bar, 20 μm. (F) Image J was used to calculate H3K9Me2/3 fluorescence intensity and nucleus size. PML‐NB numbers were counted. (G) Quantitative RT‐PCR analysis of expression of genes associated with nuclear configuration, *CBX5, LMNA, LMNB1*. (H) Quantitative RT‐PCR analysis of expression of genes responsible for immunomodulation, *IDO, TSG6, CXCL10, IL‐6*. All data are presented as mean SEM. Significance (analysed with one‐way ANNOVA) is indicated by: ns *p* > 0.05, *p < 0.05, **p < 0.01, ***p < 0.001, ****p < 0.001.

We next investigated whether the changes in nuclear architecture induced by inflammatory cytokines are irreversible by withdrawing the inflammatory cytokines after treatment for 24 h (Figure 8A). We found that the proliferation rate of hMSCs significantly recovered 1 day after withdrawal, and the cells were able to continue their normal proliferation (Figure 8B). Correspondingly, the expression level of *CDKN1A* was decreased with the recovery of cell proliferation (Figure 8C). While the inflammatory cytokines enlarged hMSCs and led to the loss of vimentin, the hMSCs gradually resumed their normal shape after the withdrawl of the inflammatory cytokines (Figure 8D). While PML‐NBs remained at a high number 1 day after the cytokines withdrawal, they gradually diminished and were significantly reduced 3 days after the inflammatory cytokine withdrawal, which was accompanied by a recovery of the H3K9Me2/3 level. The nuclei also returned to their normal size (Figure 8E,F). The expression changes of *CBX5* were consistent with the trend of H3K9Me2/3. The *LMNA* and *LMNB1* transcripts showed a similar trend (Figure 8G). Importantly, with the resumption of cell cycle progression and the restoration of nuclear configuration to the orginal state, the expression of immunoregulatory genes, such as *IDO* and *CXCL10*, in hMSCs greatly subdued 3 days after cytokine withdrawal (Figure 8H). The expression of *TSG6* and *IL‐6* dropped to pre‐stimulation level even 1 day after cytokine withdrawal. These results indicate that the loosening of nuclear architecture, inhibition of cell proliferation, cytoskeleton changes, and the induction of immunoregulatory molecules that occur in hMSCs upon inflammatory stimulation are transient responses and are reversible once the stimuli are removed.

Given the fact that cell proliferation was inhibited when hMSCs were treated with the two cytokines, and the inhibition of proliferation was accompanied by nuclear loosening. We next tested whether cell proliferation would be restored if nuclear enlargement is inhibited by the knockdown of PML. We found that although nuclear loosening caused by cytokines was inhibited by PML knockdown, the hMSCs were still inhibited in their proliferation inhibition (Figure S2), indicating that blocking nuclear loosening may not necessarily restore cell proliferation in the presence of cytokines.

## 4 DISCUSSION

In response to inflammatory stimuli, MSCs undergo drastic changes in gene expression, secretome, and other phenotypes to function as potent immunoregulators and to orchestrate the resolution of inflammation and restoration of tissue homeostasis. We here report that accompanying the drastic upregulation of a large number of effector molecules of immunomodulation in response to stimulation by inflammatory cytokines, hMSCs exhibt a reduced proliferation rate and are greatly altered in nuclear configuration, including enlargement of nucleus, thinning of nuclear envelope, decreased expression of nuclear laminins, and decondensation of heterochromatin. These features resemble those in senescent cells. The nuclear conformational changes are closely related to the massive increase in transcription required for hMSCs to perform immunomodulatory and reparatory functions in an inflammatory environment of damaged tissues. Furthermore, we have demonstrated a novel role for PML‐NB in regulating the nuclear configuration in cytokine‐stimulated hMSCs. The cytokine‐induced nuclear reconfiguration was attenuated in hMSCs when PML was depleted, which was accompanied by a reduced expression of immunomodulators by hMSCs. The hMSCs lacking PML were consequently compromised in their anti‐inflammatory functions.

We observed that with the acquisition of the greatly augmented immunomodulatory function upon exposure to the two cytokines, the hMSCs was greatly inhibited in their proliferation, but resumed their propagation rate after the cytokines were removed. Meanwhile, the immunomodulatory effectors were downregulated with the recovery of cell proliferation. Therefore it appears that the immunomodulatory function and cell propagation are mutually exclusive. It is possible that to allow for a massive output of immunomodulatory effectors, the MSCs need to stop or slow down cell division so that more resources are allocated to biosynthesis of the great variety of cytokines, chemokines and other effectors. Indeed, some effectors can be upregulated up to one‐million‐fold.^15^ Preparation and execution of cell division will likely divert the resources and energy that are in high demand for the biosynthesis of the large variety of effector molecules in large quantities.

Heterochromatin is believed to function as a barrier to transcription factors and contributes to the determination and maintenance of cell identity.^44^, ^45^ It is interesting to note that DNA hypomethylation or histone modifications that are associated with increased transcriptional activities usually lead to increased immune cell responses, including increased expression of interferons, and contribute to autoimmune diseases and success of ant‐tumour immunotherapy.^46^, ^47^, ^48^, ^49^, ^50^, ^51^, ^52^ Such epigenetic modifications frequently result in aberrant transcription of repetitive DNA elements or retrotransposons, which can elicit an interferon response. It is possible that the transcripts unleashed by chromatin loosening may function to reinforce the function of interferon. While the transcription of repetitive DNA elements and retrotransposons invariably augments immune responses in immune cells, epithelial cells and cancer cells, the chromatin loosening instead confers MSCs greatly enhanced immunosuppressive function. In this context, MSCs appear to function as a neutralizer or stabilizer to counterbalance the immune response and maintain tissue homeostasis.

PML has been documented to drive cellular senescence in multiple studies.^19^, ^20^, ^21^, ^22^, ^23^ The mechanisms underlying the PML‐induced senescence include activation of p53,^19^ regulation of E2Fs,^21^ and repression of TBX2.^22^ Although PML was also reported to regulate the dynamics of heterochromatin,^53^, ^54^ the physiological relevance is not clear. In particular, whether PML contributes to cellular senescence by regulating nuclear configuration and chromatin dynamics has not been defined. We observed that the cytokine‐stimulated hMSCs resemble senescent cells in many aspects, including reduced proliferation, enlarged nuclei, loss of lamin B and A/C, and decondensation of heterochromatin. However, the senescence‐like features in hMSCs in response to inflammatory stimulation are transient and are dependent on the continuous presence of the inflammatory cytokines. It is possible that cellular senescence as well as the senescence‐like phenotypes described here may each represent adaptive response to stress. The heterochromatin reconfiguration may serve to sustain the SASP in senescent cells and the secretome in cytokine‐induced hMSCs respectively. Both phenotypes require massive biosynthesis, though not for fast proliferation. Interestingly, the acquisition of these senescence‐like features by hMSCs depends on PML. Whether the nuclear reconfiguration and SASP in senescent cells also require PML remains to be determined.

## AUTHOR CONTRIBUTIONS

Changshun Shao, Yufang Shi, Yunpeng Chu, conception and design of the study. Yunpeng Chu and Zishan Jiang, conduction of the experiments, data collection and analysis, manuscript preparation. Zheng Gong, Xiaocao Ji, Mengting Zhu and Pixia Gong, conduction of the animal experiments. Changshun Shao and Yufang Shi interpreted the data and wrote the manuscript. Qianwen Shang, Lijuan Cao, Yongjing Chen and Peishan Li made significant intellectual contribution.

## CONFLICT OF INTEREST STATEMENT

The authors declare that they have no known competing financial interests or personal relationships that could influence the work reported in this paper.

## Supporting information


**Figure S1.** The impact of inflammatory cytokines on mouse bone marrow‐derived MSCs and various human cell lines in terms of nuclear configuration. (A) Immunofluorescence analysis was conducted to assess nucleus size, focusing on H3K9Me2/3 and H3K9Me2/3 expression levels in control mouse MSCs as well as mMSCs pretreated with IFNγ and TNFα at P9. Scale bar, 50 μm. (B–D) Immunofluorescence analysis was performed to evaluate nucleus size, focus on LMNA/C and HP1α expression in different cell lines with or without IFNγ and TNFα treatment. These cell lines include: (B) U4A; (C) Hep G2; (D) EA.hy926. Scale bar, 50 μm.
**Figure S2.** PML does not influence the inflammatory cytokines‐induced decrease in cell proliferation. (A) Flow cytometry analysis of EDU after PML knockdown and then treated with IFNγ and TNFα in hMSCs.

Table S1:

## Data Availability

Data sharing is not applicable to this article as no new data were created or analyzed in this study.

## References

[1] Gao L , Wu K , Liu Z , et al. Chromatin accessibility landscape in human early embryos and its association with evolution. Cell. 2018;173(1):248‐259. e15. 29526463 10.1016/j.cell.2018.02.028

[2] Klemm SL , Shipony Z , Greenleaf WJ . Chromatin accessibility and the regulatory epigenome. Nat Rev Genet. 2019;20(4):207‐220. 30675018 10.1038/s41576-018-0089-8

[3] Gorkin DU , Barozzi I , Zhao Y , et al. An atlas of dynamic chromatin landscapes in mouse fetal development. Nature. 2020;583(7818):744‐751. 32728240 10.1038/s41586-020-2093-3 PMC7398618

[4] Misteli T . The self‐organizing genome: principles of genome architecture and function. Cell. 2020;183(1):28‐45. 32976797 10.1016/j.cell.2020.09.014 PMC7541718

[5] Strom AR , Emelyanov AV , Mir M , Fyodorov DV , Darzacq X , Karpen GH . Phase separation drives heterochromatin domain formation. Nature. 2017;547(7662):241‐245. 28636597 10.1038/nature22989 PMC6022742

[6] Kinchen J , Chen HH , Parikh K , et al. Structural remodeling of the human colonic mesenchyme in inflammatory bowel disease. Cell. 2018;175(2):372‐386. e17. 30270042 10.1016/j.cell.2018.08.067 PMC6176871

[7] Lee JH , Tammela T , Hofree M , et al. Anatomically and functionally distinct lung mesenchymal populations marked by Lgr5 and Lgr6. Cell. 2017;170(6):1149‐1163. e12. 28886383 10.1016/j.cell.2017.07.028 PMC5607351

[8] Zepp JA , Zacharias WJ , Frank DB , et al. Distinct mesenchymal lineages and niches promote epithelial self‐renewal and Myofibrogenesis in the lung. Cell. 2017;170(6):1134‐1148. e10. 28886382 10.1016/j.cell.2017.07.034 PMC5718193

[9] Zheng Z , Li YN , Jia S , et al. Lung mesenchymal stromal cells influenced by Th2 cytokines mobilize neutrophils and facilitate metastasis by producing complement C3. Nat Commun. 2021;12(1):6202. 34707103 10.1038/s41467-021-26460-z PMC8551331

[10] Roulis M , Kaklamanos A , Schernthanner M , et al. Paracrine orchestration of intestinal tumorigenesis by a mesenchymal niche. Nature. 2020;580(7804):524‐529. 32322056 10.1038/s41586-020-2166-3 PMC7490650

[11] Shi Y , du L , Lin L , Wang Y . Tumour‐associated mesenchymal stem/stromal cells: emerging therapeutic targets. Nat Rev Drug Discov. 2017;16(1):35‐52. 27811929 10.1038/nrd.2016.193

[12] Shi Y , Wang Y , Li Q , et al. Immunoregulatory mechanisms of mesenchymal stem and stromal cells in inflammatory diseases. Nat Rev Nephrol. 2018;14(8):493‐507. 29895977 10.1038/s41581-018-0023-5

[13] Han Y , Yang J , Fang J , et al. The secretion profile of mesenchymal stem cells and potential applications in treating human diseases. Signal Transduct Target Ther. 2022;7(1):92. 35314676 10.1038/s41392-022-00932-0 PMC8935608

[14] Wang Y , Fang J , Liu B , Shao C , Shi Y . Reciprocal regulation of mesenchymal stem cells and immune responses. Cell Stem Cell. 2022;29(11):1515‐1530. 36332569 10.1016/j.stem.2022.10.001

[15] Ren G , Zhang L , Zhao X , et al. Mesenchymal stem cell‐mediated immunosuppression occurs via concerted action of chemokines and nitric oxide. Cell Stem Cell. 2008;2(2):141‐150. 18371435 10.1016/j.stem.2007.11.014

[16] Ren G , Su J , Zhang L , et al. Species variation in the mechanisms of mesenchymal stem cell‐mediated immunosuppression. Stem Cells. 2009;27(8):1954‐1962. 19544427 10.1002/stem.118

[17] Li Y , Ma X , Wu W , Chen Z , Meng G . PML nuclear body biogenesis, carcinogenesis, and targeted therapy. Trends Cancer. 2020;6(10):889‐906. 32527650 10.1016/j.trecan.2020.05.005

[18] Corpet A , Kleijwegt C , Roubille S , et al. PML nuclear bodies and chromatin dynamics: catch me if you can! Nucleic Acids Res. 2020;48(21):11890‐11912. 33068409 10.1093/nar/gkaa828 PMC7708061

[19] Pearson M , Carbone R , Sebastiani C , et al. PML regulates p53 acetylation and premature senescence induced by oncogenic Ras. Nature. 2000;406(6792):207‐210. 10910364 10.1038/35018127

[20] Ferbeyre G , de Stanchina E , Querido E , Baptiste N , Prives C , Lowe SW . PML is induced by oncogenic ras and promotes premature senescence. Genes Dev. 2000;14(16):2015‐2027. 10950866 PMC316863

[21] Vernier M , Bourdeau V , Gaumont‐Leclerc MF , et al. Regulation of E2Fs and senescence by PML nuclear bodies. Genes Dev. 2011;25(1):41‐50. 21205865 10.1101/gad.1975111 PMC3012935

[22] Martin N , Benhamed M , Nacerddine K , et al. Physical and functional interaction between PML and TBX2 in the establishment of cellular senescence. EMBO J. 2012;31(1):95‐109. 22002537 10.1038/emboj.2011.370 PMC3252579

[23] Wu HC , Rérolle D , Berthier C , et al. Actinomycin D targets NPM1c‐primed mitochondria to restore PML‐driven senescence in AML therapy. Cancer Discov. 2021;11(12):3198‐3213. 34301789 10.1158/2159-8290.CD-21-0177 PMC7612574

[24] Banani SF , Rice AM , Peeples WB , et al. Compositional control of phase‐separated cellular bodies. Cell. 2016;166(3):651‐663. 27374333 10.1016/j.cell.2016.06.010 PMC4967043

[25] Berchtold D , Battich N , Pelkmans L . A systems‐level study reveals regulators of membrane‐less organelles in human cells. Mol Cell. 2018;72(6):1035‐1049. e5. 30503769 10.1016/j.molcel.2018.10.036

[26] Delbarre E , Ivanauskiene K , Küntziger T , Collas P . DAXX‐dependent supply of soluble (H3.3‐H4) dimers to PML bodies pending deposition into chromatin. Genome Res. 2013;23(3):440‐451. 23222847 10.1101/gr.142703.112 PMC3589533

[27] Xu S , Wang S , Xing S , et al. KDM5A suppresses PML‐RARalpha target gene expression and APL differentiation through repressing H3K4me2. Blood Adv. 2021;5(17):3241‐3253. 34448811 10.1182/bloodadvances.2020002819 PMC8525237

[28] Luciani JJ , Depetris D , Usson Y , et al. PML nuclear bodies are highly organised DNA‐protein structures with a function in heterochromatin remodelling at the G2 phase. J Cell Sci. 2006;119(Pt 12:2518‐2531. 16735446 10.1242/jcs.02965

[29] Chelbi‐Alix MK , Pelicano L , Quignon F , et al. Induction of the PML protein by interferons in normal and APL cells. Leukemia. 1995;9(12):2027‐2033. 8609713

[30] Grotzinger T , Jensen K , Will H . The interferon (IFN)‐stimulated gene Sp100 promoter contains an IFN‐gamma activation site and an imperfect IFN‐stimulated response element which mediate type I IFN inducibility. J Biol Chem. 1996;271(41):25253‐25260. 8810287 10.1074/jbc.271.41.25253

[31] Wang G , Cao K , Liu K , et al. Kynurenic acid, an IDO metabolite, controls TSG‐6‐mediated immunosuppression of human mesenchymal stem cells. Cell Death Differ. 2018;25(7):1209‐1223. 29238069 10.1038/s41418-017-0006-2 PMC6030103

[32] Ding Y , Gong P , Jiang J , et al. Mesenchymal stem/stromal cells primed by inflammatory cytokines alleviate psoriasis‐like inflammation via the TSG‐6‐neutrophil axis. Cell Death Dis. 2022;13(11):996. 36433947 10.1038/s41419-022-05445-w PMC9700741

[33] Shang Q , Chu Y , Li Y , et al. Adipose‐derived mesenchymal stromal cells promote corneal wound healing by accelerating the clearance of neutrophils in cornea. Cell Death Dis. 2020;11(8):707. 32848141 10.1038/s41419-020-02914-y PMC7450061

[34] Braumüller H , Wieder T , Brenner E , et al. T‐helper‐1‐cell cytokines drive cancer into senescence. Nature. 2013;494(7437):361‐365. 23376950 10.1038/nature11824

[35] Zhang W , Li J , Suzuki K , et al. Aging stem cells. A Werner syndrome stem cell model unveils heterochromatin alterations as a driver of human aging. Science. 2015;348(6239):1160‐1163. 25931448 10.1126/science.aaa1356 PMC4494668

[36] Bedrosian TA , Houtman J , Eguiguren JS , et al. Lamin B1 decline underlies age‐related loss of adult hippocampal neurogenesis. EMBO J. 2021;40(3):e105819. 33300615 10.15252/embj.2020105819 PMC7849303

[37] Matias I , Diniz LP , Damico IV , et al. Loss of lamin‐B1 and defective nuclear morphology are hallmarks of astrocyte senescence in vitro and in the aging human hippocampus. Aging Cell. 2022;21(1):e13521. 34894056 10.1111/acel.13521 PMC8761005

[38] bin Imtiaz MK , Jaeger BN , Bottes S , et al. Declining lamin B1 expression mediates age‐dependent decreases of hippocampal stem cell activity. Cell Stem Cell. 2021;28(5):967‐977.e8. e8. 33631115 10.1016/j.stem.2021.01.015

[39] Sladitschek‐Martens HL , Guarnieri A , Brumana G , et al. YAP/TAZ activity in stromal cells prevents ageing by controlling cGAS‐STING. Nature. 2022;607(7920):790‐798. 35768505 10.1038/s41586-022-04924-6 PMC7613988

[40] Hu H , Ji Q , Song M , et al. ZKSCAN3 counteracts cellular senescence by stabilizing heterochromatin. Nucleic Acids Res. 2020;48(11):6001‐6018. 32427330 10.1093/nar/gkaa425 PMC7293006

[41] Deng L , Ren R , Liu Z , et al. Stabilizing heterochromatin by DGCR8 alleviates senescence and osteoarthritis. Nat Commun. 2019;10(1):3329. 31350386 10.1038/s41467-019-10831-8 PMC6659673

[42] Delbarre E , Janicki SM . Modulation of H3.3 chromatin assembly by PML: a way to regulate epigenetic inheritance. Bioessays. 2021;43(10):e2100038. 34423467 10.1002/bies.202100038

[43] Salsman J , Rapkin LM , Margam NN , Duncan R , Bazett‐Jones DP , Dellaire G . Myogenic differentiation triggers PML nuclear body loss and DAXX relocalization to chromocentres. Cell Death Dis. 2017;8(3):e2724. 28358373 10.1038/cddis.2017.151 PMC5386546

[44] Becker JS , Nicetto D , Zaret KS . H3K9me3‐Dependent Heterochromatin: Barrier to Cell Fate Changes. Trends Genet. 2016;32(1):29‐41. 26675384 10.1016/j.tig.2015.11.001 PMC4698194

[45] McCarthy RL , Zhang J , Zaret KS . Diverse heterochromatin states restricting cell identity and reprogramming. Trends Biochem Sci. 2023;48:513‐526. 36990958 10.1016/j.tibs.2023.02.007 PMC10182259

[46] Absher DM , Li X , Waite LL , et al. Genome‐wide DNA methylation analysis of systemic lupus erythematosus reveals persistent hypomethylation of interferon genes and compositional changes to CD4+ T‐cell populations. PLoS Genet. 2013;9(8):e1003678. 23950730 10.1371/journal.pgen.1003678 PMC3738443

[47] Leonova K , Safina A , Nesher E , et al. TRAIN (Transcription of repeats activates INterferon) in response to chromatin destabilization induced by small molecules in mammalian cells. Elife. 2018;7:7. 10.7554/eLife.30842 PMC5815852 29400649

[48] Ding W , Pu W , Wang L , et al. Genome‐wide DNA methylation analysis in systemic sclerosis reveals hypomethylation of IFN‐associated genes in CD4(+) and CD8(+) T cells. J Invest Dermatol. 2018;138(5):1069‐1077. 29248544 10.1016/j.jid.2017.12.003

[49] Sheng W , LaFleur MW , Nguyen TH , et al. LSD1 ablation stimulates anti‐tumor immunity and enables checkpoint blockade. Cell. 2018;174(3):549‐563. e19. 29937226 10.1016/j.cell.2018.05.052 PMC6063761

[50] Wang Y , Xie H , Chang X , et al. Single‐cell dissection of the multiomic landscape of high‐grade serous ovarian cancer. Cancer Res. 2022;82(21):3903‐3916. 35969151 10.1158/0008-5472.CAN-21-3819 PMC9627134

[51] Beck MA , Fischer H , Grabner LM , et al. DNA hypomethylation leads to cGAS‐induced autoinflammation in the epidermis. EMBO J. 2021;40(22):e108234. 34586646 10.15252/embj.2021108234 PMC8591534

[52] Rajshekar S , Yao J , Arnold PK , et al. Pericentromeric hypomethylation elicits an interferon response in an animal model of ICF syndrome. Elife. 2018;7:7. 10.7554/eLife.39658 PMC6261255 30484769

[53] Delbarre E , Ivanauskiene K , Spirkoski J , et al. PML protein organizes heterochromatin domains where it regulates histone H3.3 deposition by ATRX/DAXX. Genome Res. 2017;27(6):913‐921. 28341773 10.1101/gr.215830.116 PMC5453325

[54] Shastrula PK , Sierra I , Deng Z , et al. PML is recruited to heterochromatin during S phase and represses DAXX‐mediated histone H3.3 chromatin assembly. J Cell Sci. 2019;132(6):jcs220970. 30796101 10.1242/jcs.220970 PMC6451418

