# Supplementary material for: PML‐mediated nuclear loosening permits immunomodulation of mesenchymal stem/stromal cells under inflammatory conditions
Source: Cell Prolif. 2023 Oct 20;57(4):e13566. doi: 10.1111/cpr.13566 (PMC10984101; doi:10.1111/cpr.13566)
Supplement: Supplementary file 1 — Figure S1. The impact of inflammatory cytokines on mouse bone marrow‐derived MSCs and various human cell lines in terms of nuclear configuration. (A) Immunofluorescence analysis was conducted to assess nucleus size, focusing on H3K9Me2/3 and H3K9Me2/3 expression levels in control mouse MSCs as well as mMSCs pretreated with IFNγ and TNFα at P9. Scale bar, 50 μm. (B–D) Immunofluorescence analysis was performed to evaluate nucleus size, focus on LMNA/C and HP1α expression in different cell lines with or without IFNγ and TNFα treatment. These cell lines include: (B) U4A; (C) Hep G2; (D) EA.hy926. Scale bar, 50 μm. Figure S2. PML does not influence the inflammatory cytokines‐induced decrease in cell proliferation. (A) Flow cytometry analysis of EDU after PML knockdown and then treated with IFNγ and TNFα in hMSCs. [file CPR-57-e13566-s001.pdf]

Supplementary Figure 1.

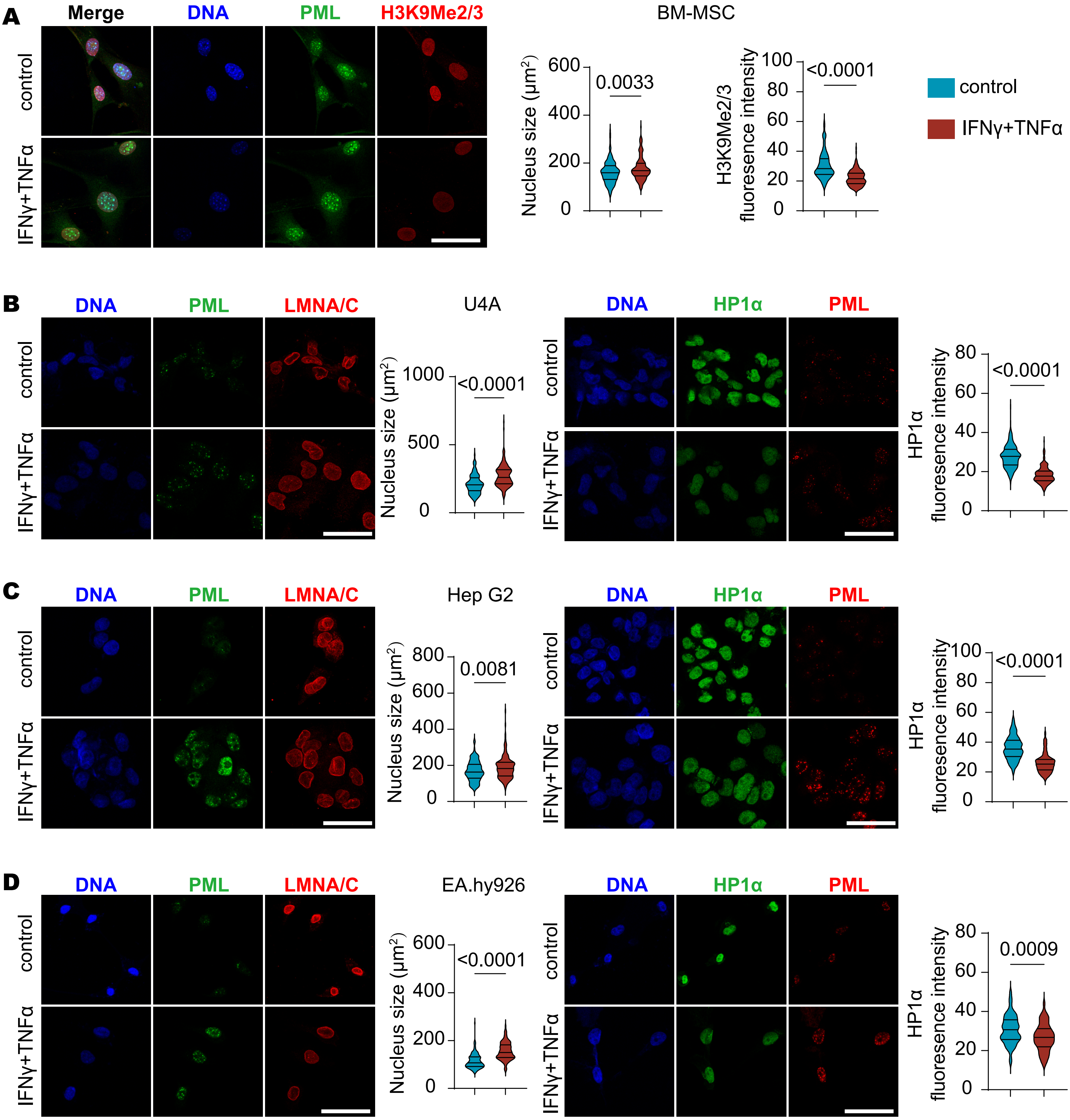

Supplementary Figure 1. The impact of inflammatory cytokines on mouse bone marrow-derived MSCs and various human cell lines in terms of nuclear configuration.

(A) Immunofluorescence analysis was conducted to assess nucleus size, focusing on H3K9Me2/3 and H3K9Me2/3 expression levels in control mouse MSCs as well as mMSCs pretreated with IFNγ and TNFα at P9. Scale bar, 50 μm. (B-D) Immunofluorescence analysis was performed to evaluate nucleus size, focus on LMNA/C and HP1α expression in different cell lines with or without IFNγ and TNFα treatment. These cell lines include: B) U4A; C) Hep G2;D) EA.hy926. Scale bar, 50 μm.

Supplementary Figure 2.

A

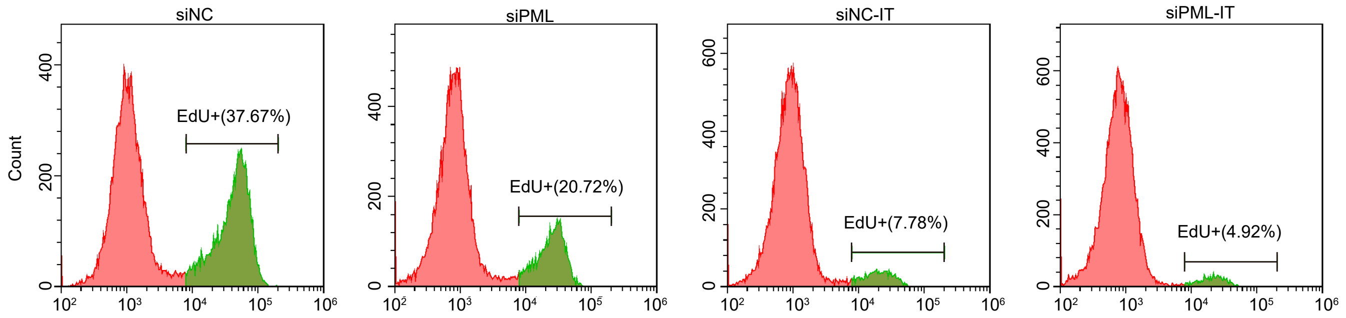

Supplementary Figure 2. PML does not influence the inflammatory cytokines-induced decrease in cell proliferation. (A) Flow cytometry analysis of EDU after PML knockdown and then treated with IFNγ and TNFα in hMSCs.
